# Supplementary material for: Phylogenetic Relationships among TnpB-Containing Mobile Elements in Six Bacterial Species
Source: Genes (Basel). 2023 Feb 19;14(2):523. doi: 10.3390/genes14020523 (PMC9956272; doi:10.3390/genes14020523)
Supplement: Supplementary file 1 [file genes-14-00523-s001.zip › Supplementary Figure.pdf]

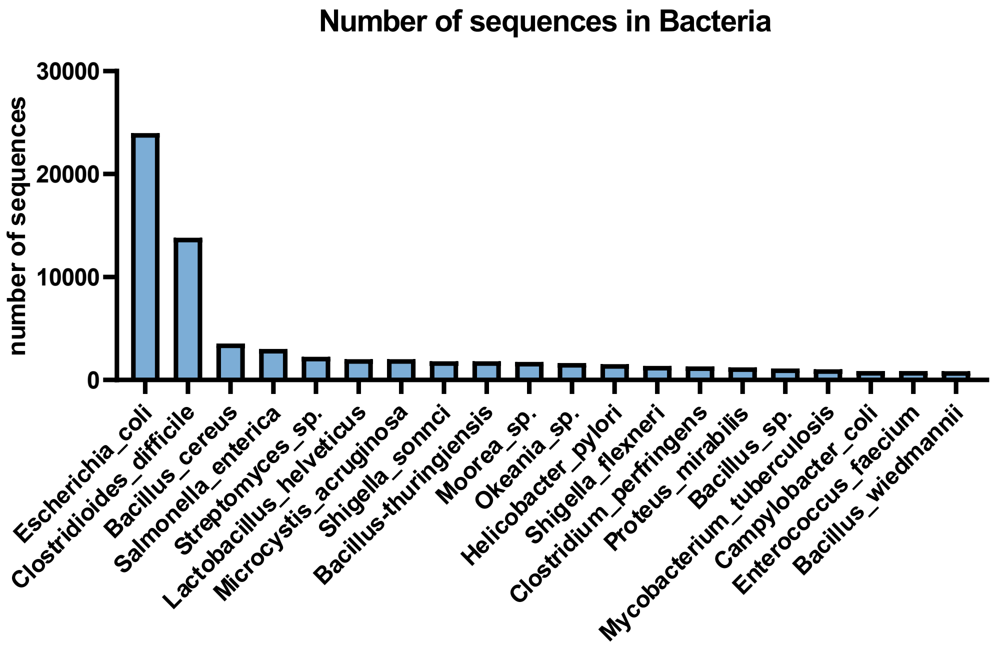

Supplementary Figure. S1. The number of TnpB hits in 19 species. The figure listed the top 19 species containing TnpB hits.

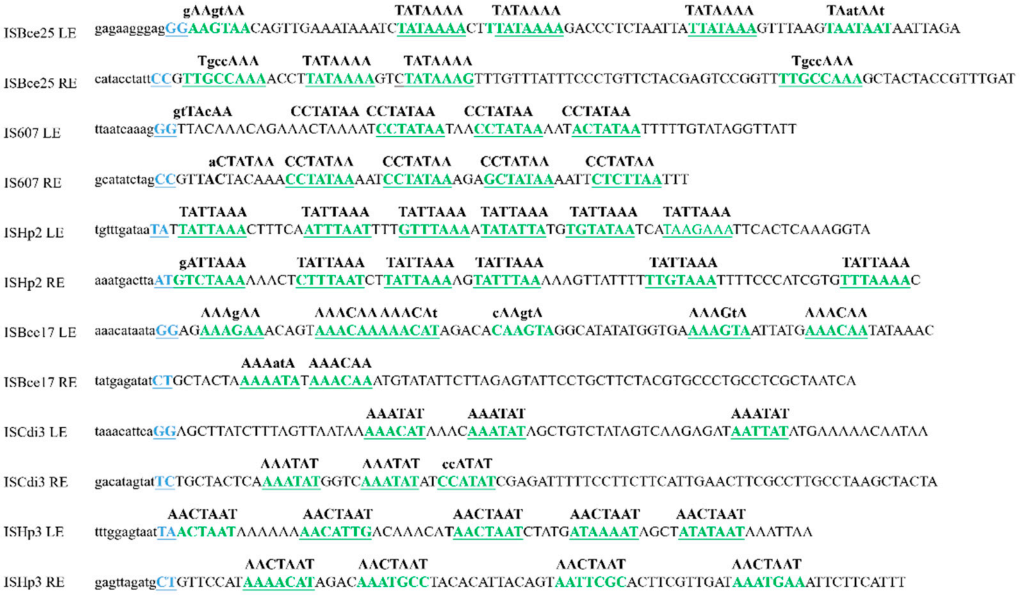

Supplementary Figure. S2. The LE and RE structures of the *IS607* group. The cleavage sites are shown in blue. The direct repeat sequences are shown in green. The top nucleotide sequences show variations from the direct repeat sequences.

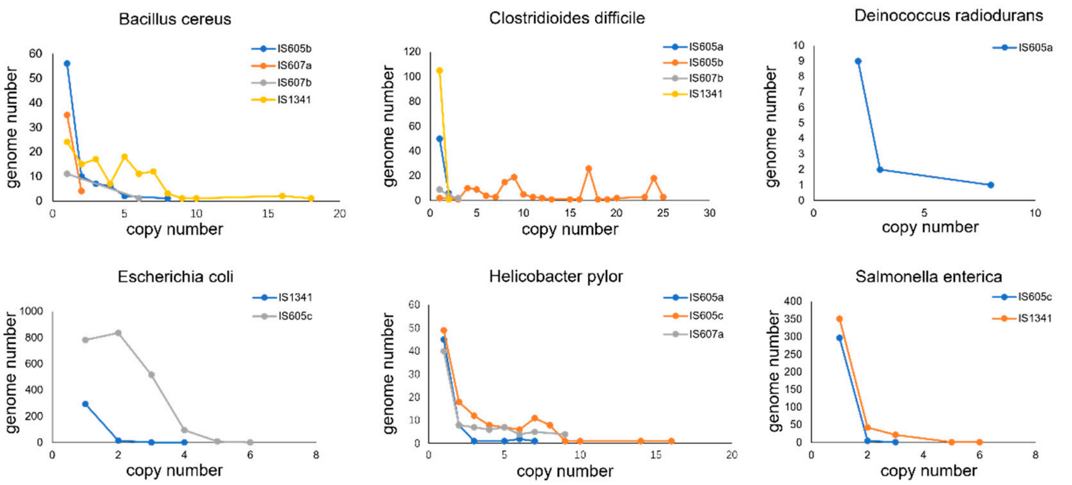

Supplementary Figure. S3.The various copy number of IS elements in different genomes. The most of genomes only contains one copy, and a small fraction contains multiple copies..

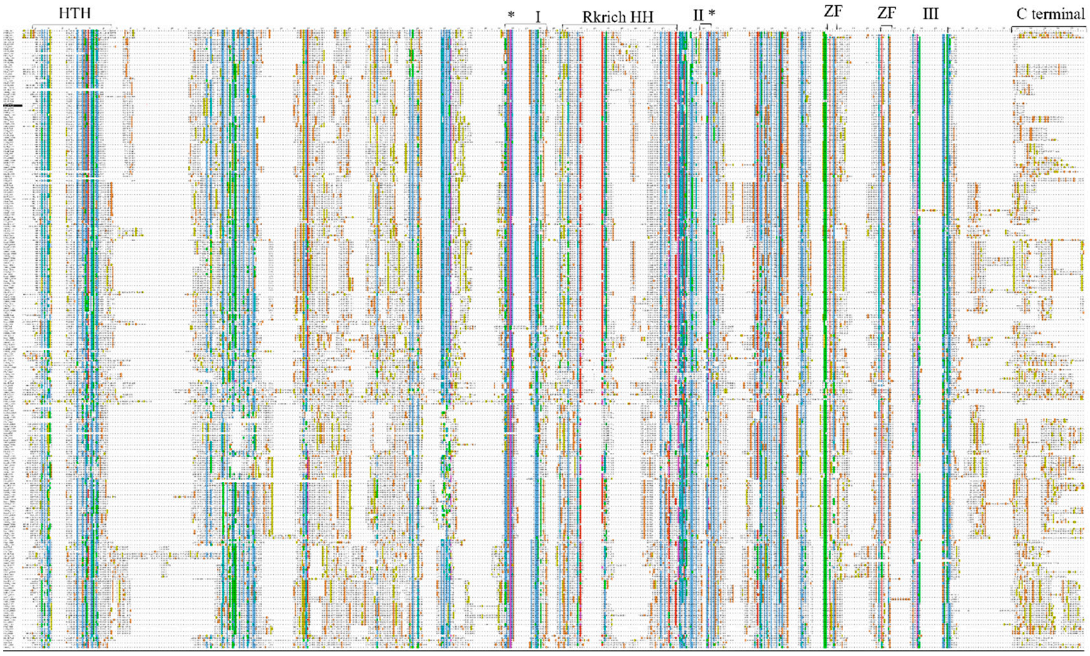

Supplementary Figure. S4. Alignment of 274 TnpB sequences includes represented sequences from ISfinder database and 39-mined sequence from six species.

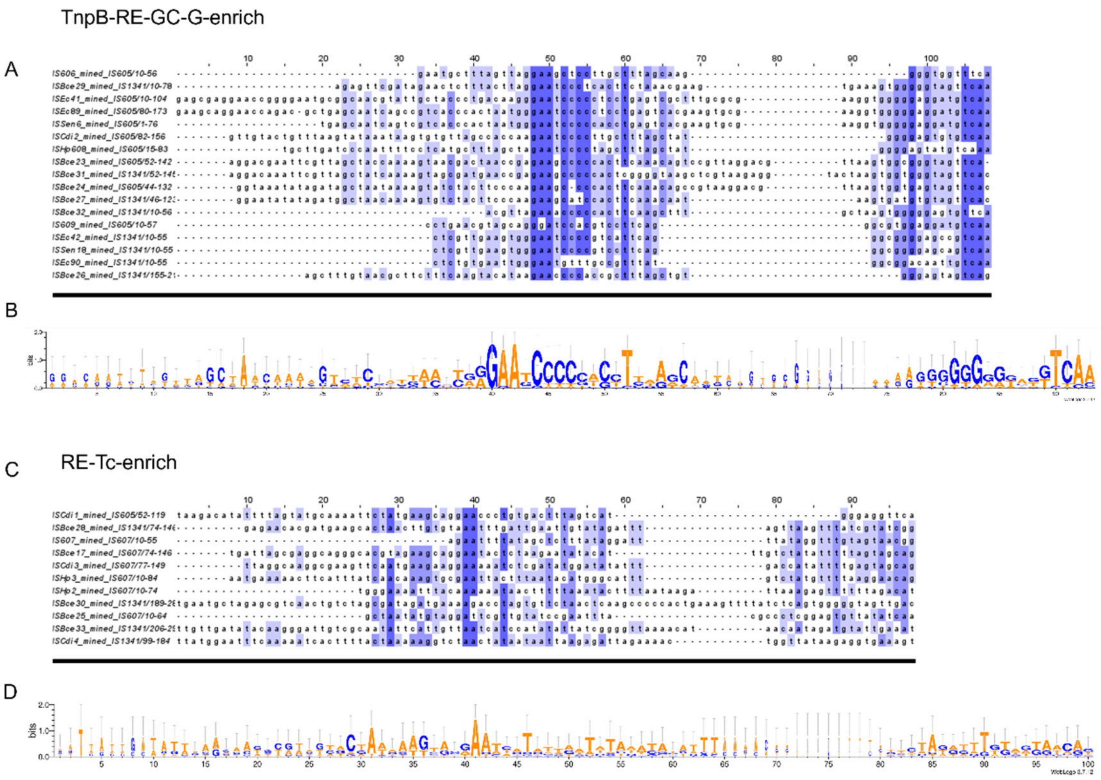

Supplementary Figure. S5. The character of RE sequences. (A) The alignment of part of TnpB-mined RE sequences. Two motifs were found which showed GC-rich position. (B) The logo of this type RE showed C-rich and G-rich conserved motifs. (C) The alignment of AT-TG-rich type RE sequences which showed two conserved motifs. (D) The logo of AT-TG -rich RE. This type RE showed one TA-rich motif and one TG-rich motif.
