# Supplementary material for: Phylogenetic Relationships among TnpB-Containing Mobile Elements in Six Bacterial Species
Source: Genes (Basel). 2023 Feb 19;14(2):523. doi: 10.3390/genes14020523 (PMC9956272; doi:10.3390/genes14020523)
Supplement: Supplementary file 1 [file genes-14-00523-s001.zip › Supplementuaty File S1.pdf]

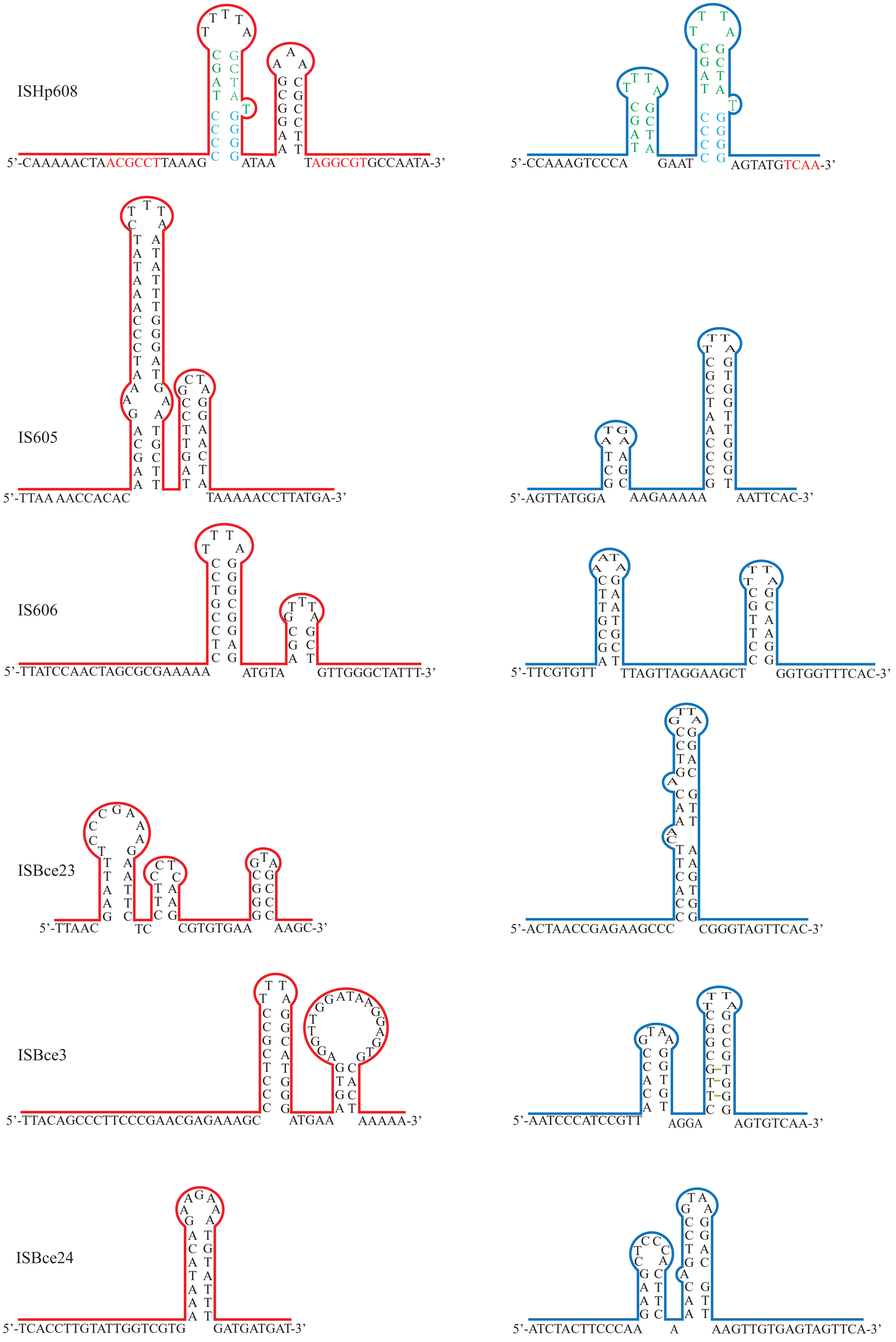

ISHp608
5'-CAAAAACTAACGCCTTAAAG
ATAA
TAGGCGTGCCAATA-3'
5'-CCAAAGTCCCA
GAAT
AGTATGTCAA-3'
IS605
5'-TTAA AACCACAC
TAAAAACCTTATGA-3'
5'-AGTTATGGA
AAGAAAAA
AATTCAC-3'
IS606
5'-TTATCCAACTAGCGCGAAAAA
ATGTA
GTTGGGCTATTT-3'
5'-TTCGTGTT
TTAGTTAGGAAGCT
GGTGGTTTCAC-3'
ISBce23
5'-TTAAC
TC
CGTGTGAA
AAGC-3'
5'-ACTAACCGAGAAGCCC
CGGGTAGTTCAC-3'
ISBce3
5'-TTACAGCCCTTCCCGAACGAGAAAGC
ATGAA
AAAAA-3'
5'-AATCCCATCCGTT
AGGA
AGTGTCAA-3'
ISBce24
5'-TCACCTTGTATTGGTCGTG
GATGATGAT-3'
5'-ATCTACTTCCCAA
A
AAGTTGTGAGTAGTTCA-3'

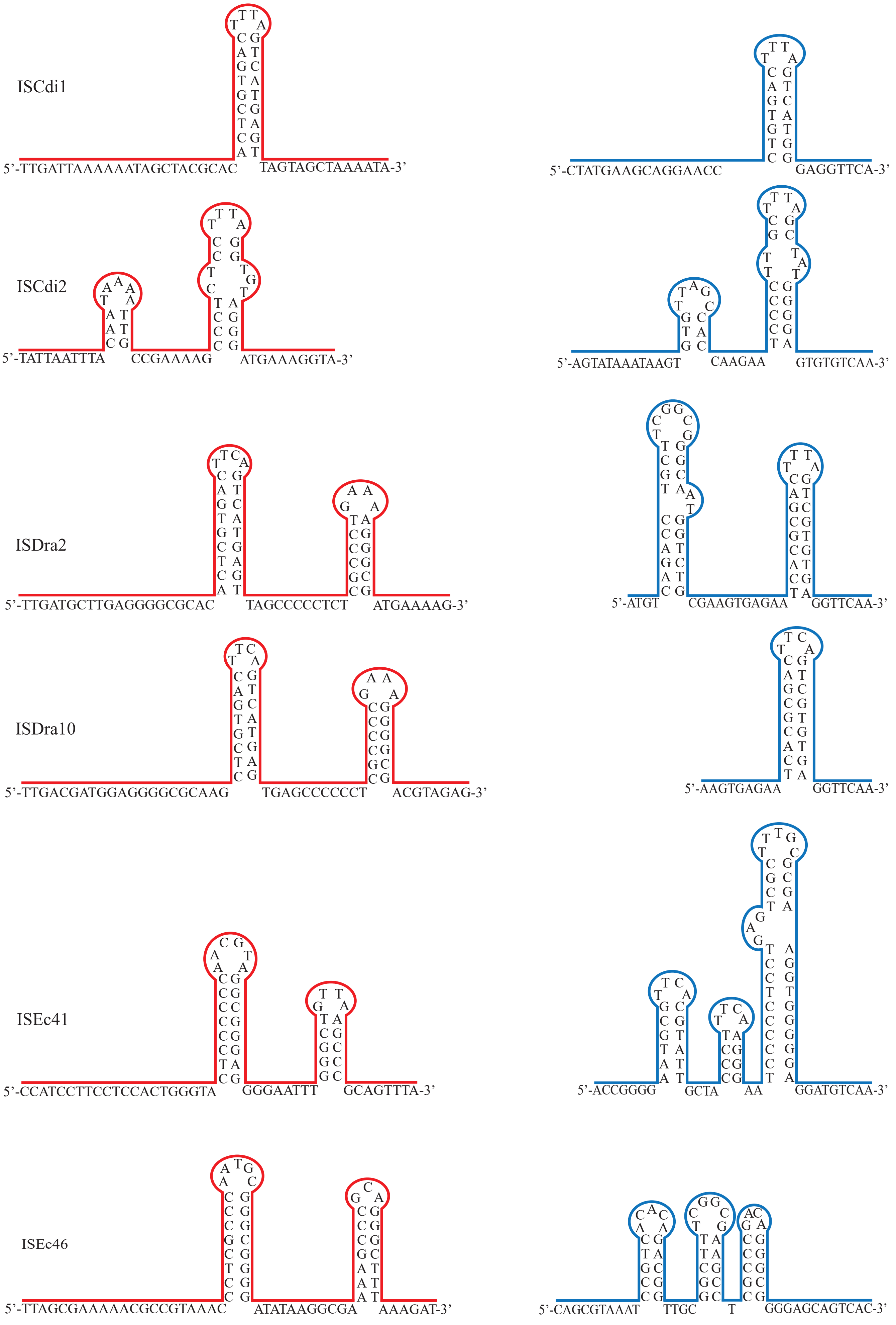

ISCdi1
5'-TTGATTAAAAAATAGCTACGCAC
TAGTAGCTAAAATA-3'
5'-CTATGAAGCAGGAACC
GAGGTTCA-3'
ISCdi2
5'-TATTAATTTA
CCGAAAAG
ATGAAAGGTA-3'
5'-AGTATAAATAAGT
CAAGAA
GTGTGTCAA-3'
ISDra2
5'-TTGATGCTTGAGGGGCGCAC
TAGCCCCCTCT
ATGAAAAG-3'
5'-ATGT
CGAAGTGAGAA
GGTTCAA-3'
ISDra10
5'-TTGACGATGGAGGGGCGCAAG
TGAGCCCCCCT
ACGTAGAG-3'
5'-AAGTGAGAA
GGTTCAA-3'
ISEc41
5'-CCATCCTTCCTCCACTGGGTA
GGGAATTT
GCAGTTTA-3'
5'-ACCGGGG
GCTA
AA
GGATGTCAA-3'
ISEc46
5'-TTAGCGAAAAACGCCGTAAAC
ATATAAGGCGA
AAAGAT-3'
5'-CAGCGTAAAT
TTGC
T
GGGAGCAGTCAC-3'

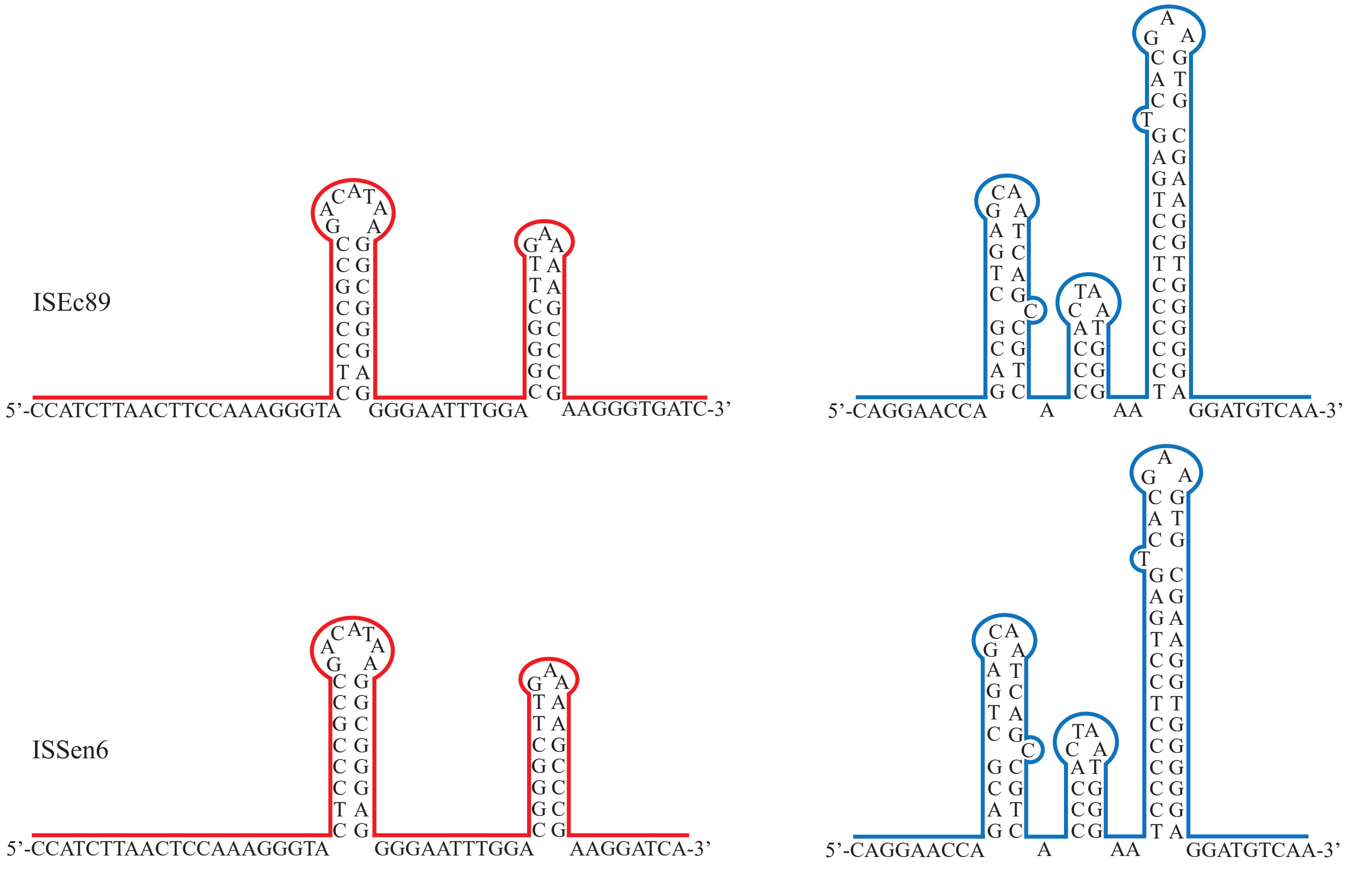

Supplementary file S1. The terminal structure of IS605 subgroup. The left ends were showed in red. The right ends were showed in blue. The hairpin structure of the terminal ends were predicted by Mfold (DNA Folding Form (mfold.org)) and (RNAfold web server (univie.ac.at)).
